# De-Orphaning the Structural Proteome through Reciprocal Comparison of Evolutionarily Important Structural Features

**DOI:** 10.1371/journal.pone.0002136

**Published:** 2008-05-07

**Authors:** R. Matthew Ward, Serkan Erdin, Tuan A. Tran, David M. Kristensen, Andreas Martin Lisewski, Olivier Lichtarge

**Affiliations:** 1 Department of Molecular and Human Genetics, Baylor College of Medicine, Houston, Texas, United States of America; 2 Graduate Program in Structural and Computational Biology and Molecular Biophysics, Baylor College of Medicine, Houston, Texas, United States of America; Wellcome Trust Centre for Human Genetics, United Kingdom

## Abstract

Function prediction frequently relies on comparing genes or gene products to search for relevant similarities. Because the number of protein structures with unknown function is mushrooming, however, we asked here whether such comparisons could be improved by focusing narrowly on the key functional features of protein structures, as defined by the Evolutionary Trace (ET). Therefore a series of algorithms was built to (a) extract local motifs (3D templates) from protein structures based on ET ranking of residue importance; (b) to assess their geometric and evolutionary similarity to other structures; and (c) to transfer enzyme annotation whenever a plurality was reached across matches. Whereas a prototype had only been 80% accurate and was not scalable, here a speedy new matching algorithm enabled large-scale searches for reciprocal matches and thus raised annotation specificity to 100% in both positive and negative controls of 49 enzymes and 50 non-enzymes, respectively—in one case even identifying an annotation error—while maintaining sensitivity (∼60%). Critically, this Evolutionary Trace Annotation (ETA) pipeline requires no prior knowledge of functional mechanisms. It could thus be applied in a large-scale retrospective study of 1218 structural genomics enzymes and reached 92% accuracy. Likewise, it was applied to all 2935 unannotated structural genomics proteins and predicted enzymatic functions in 320 cases: 258 on first pass and 62 more on second pass. Controls and initial analyses suggest that these predictions are reliable. Thus the large-scale evolutionary integration of sequence-structure-function data, here through reciprocal identification of local, functionally important structural features, may contribute significantly to de-orphaning the structural proteome.

## Introduction

The functions of most proteins solved by the Protein Structure Initiative (PSI) [1]–[3] and other structural genomics (SG) projects remain unknown [4]. One reason is that SG typically selects targets with less than 30% sequence identity to known structures [5]–[10], which limits annotation through homology. Thus eighty percent of the 630 new SG structures solved last year lack annotation, and as of May 2007 over a third of the almost 4400 structures in the PDB [11] with the “structural genomics” keyword were labeled “hypothetical” or “unknown function”.

Eventually, automated experimental screens should reveal function on a large scale [12], but for now their range of assays is limited. Analysis of gene ontology (GO) [13] annotations of the UNIPROT database [14] indicates that 98% of the 26 million annotations of 3.5 million proteins are inferred from computational methods, frequently BLAST [15] or PSI-BLAST [16]. One concern about this universal strategy [17]–[19] is that it entails errors at sequence identity below 40% [17], [20]–[23], and occasionally even above that threshold [24]–[26]. A derivative concern is that these errors may propagate [2], [27], [28]. A critical goal of annotation techniques therefore is to improve specificity.

Alternative strategies also rely on comparisons of sequence or structure, either in whole or just in part. Examples include sequence motifs [29], [30]; global fold (DALI [31], VAST [32], SSM [33], Grath [34], PDBFun [35], TOPS [36], SuMo [37], [38], CM [39]); and small structural motifs—the object of this study. In contrast to all these techniques, which seek elements of sequence or structure that are intrinsically correlated with a biological role across species, other approaches such as ProtFun [40] suggest function based on posttranslational modifications, subcellular localization, and physical/chemical properties, while still others suggest function from pyhlogenetic profiles [41], or from relationships within species that reveal genome modules [42], expression modules (CAST [43]), or physical modules [44].

The focus here is on three dimensional (3D) template methods, which search for local structural similarity of key functional residues in separate proteins [45] using methods such as geometric hashing [46]–[48]. Examples include the geometric matching of function-associated 3D templates to proteins (Jess [49], [50], Rigor [51], Pints [52], ASSAM [53], Fuzzy Functional Forms [54], geometric potential [55]); or the comparison of surface patches (3D profiles [56], [57]), clefts (Surfnet [58], VOIDOO [59], CASTp [50], SiteEngine [60], pvSOAR [61]), or binding sites (Surfnet-ConSurf [62], eF-site [63], Cavbase [64], PDBSiteScan [65], [66]). These methods often depend on experimentally identified motifs, which are relatively few [67], and can be non-specific. One important alternative approach therefore is to create templates for the protein of unknown function. Methods such as GASPS [68] use machine learning techniques, while the ProFunc metaserver's reverse templates method [69] accomplishes this through the semi-random selection of multiple small templates.

Another possibility for creating templates in the absence of experimental data on functional sites is to iteratively exploit evolutionary constraints: first to identify evolutionarily important residues that suggest 3D templates, and then to sort which of their matches are functionally relevant. For example, starting from the premise that the Evolutionary Trace (ET) can identify likely functional sites [70], [71] and their key residue determinants [72]–[75], proof of concept studies optimized the heuristic selection of 3D templates from ET residues [76] so that matches in other structures suggest functional similarity [77]. Yet, before it can be deployed on a large scale this annotation strategy still needs to be faster and more specific. This study addresses both problems. First, a new algorithm increases structural matching speed by two orders of magnitude. In turn, this makes it possible to consider all-against-all template matches and enables the addition of a new requirement for reciprocal matching. This requirement considerably increases functional annotation specificity, much as reciprocal best hits in sequence searches help identify orthologs [78], [79].

Here, the gain in annotation specificity from reciprocal matching is rooted in the fact that given two proteins S and T with respective templates *s* and *t*, then *s*≠*t* unless S and T are close homologs (and their cross-annotation trivial). As a result the search for *s* in T and for *t* in S should effectively be complementary tests, rather than redundant ones. If both turn out positive, then the possibility that the two proteins are functionally similar has more support than if only one template had matched the other protein. This study therefore tests the hypothesis that forcing the ET Annotation pipeline (ETA) to yield *reciprocal* template matches, from *t* to S, and from *s* to T, will increase annotation specificity and accuracy. Positive controls on enzymes and negative controls on non-enzymes show this is true on the small and large scales: reciprocal ETA routinely achieves better than 92% accuracy, while its increased efficiency translates into its application to all structural genomics proteins, yielding new enzymatic annotations for 320 proteins.

## Results and Discussion

### Evolutionary Trace Annotation

**Figure 1:**
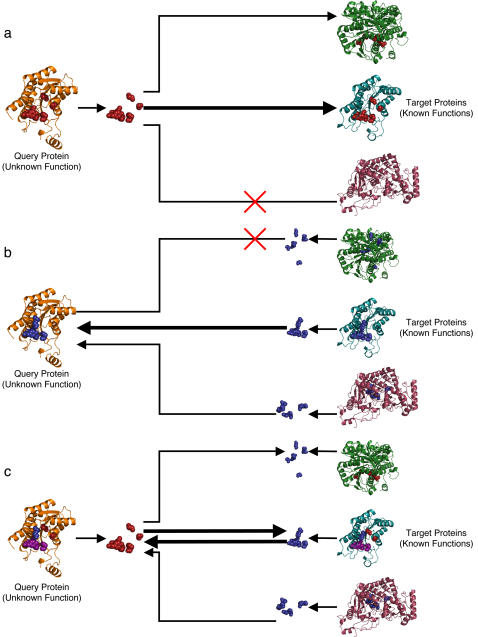
Matching Strategies. Schematic overview of the three matching strategies. 1a, one-to-many matching; 1b, many-to-one matching; 1c, the two superimposed. Lines represent template searches; arrows, matches; bold lines, correct matches; other lines, incorrect matches; X's, no match. Purple spheres are residues in both the source and target template and match; red spheres, residues in the query template and target match; blue spheres, residues in the target template and query match.

This study first set out to improve ETA's *one-to-many* annotation strategy, shown in Figure 1a (see Methods for details). In this search, ET ranks the evolutionary importance of the residues in a source protein of unknown function, *S*. Heuristics then select six residues based on their ranks, solvent accessibility, and clustering to define a 3D template denoted *s*. A geometric search then matches *s* to a set of target protein structures **T** = {*T_i_*} (Dataset S1), each with known function *f_i_*. Since a small root mean squared deviation (RMSD) alone is not sufficient to guarantee the functional relevance of a match [77], [80], a support vector machine (SVM) trained on enzymes (Dataset S2) considers in addition to RMSD whether the matches also fall on evolutionarily important regions of *T_i_*. The resulting matches *T_j_* (where the index *j* denotes matches) yield a set of possible functions **F** = {*f_j_*} of *S*, and if one function *f_0_* achieves plurality (recurs among *T_j_*'s more often than any other), then it is chosen as the single most likely annotation [76].

To enable large-scale ETA searches, the first task was to accelerate the pipeline, specifically the geometric matching algorithm. A new Paired Distance Matching (PDM) algorithm was introduced that breaks templates down into pairwise distances among alpha carbons and searches for them iteratively in target structures without considering chirality (see methods). The variability of template amino acids was also narrowed, and a strict 2 Å cutoff replaced a more flexible but slower statistical model for the maximum acceptable RMSD between a template and match. Table 1 shows that in a control set of 49 structural genomics enzymes used previously (Dataset S3), annotation accuracy edged upward from 79% to 83%. Critically, search time fell 20-fold, thereby allowing large-scale and more complex search schemes.

**Table 1:** ETA Annotation of PSI Test Set Using MA or PDM.

	MA ETA	PDM ETA
Proteins	49	49
With Matches	38/49 (78%)	32/49 (65%)
With At Least One True Match	30/38 (79%)	28/32 (88%)
With Vote Winners	28/38 (74%)	24/32 (75%)
With Correct Winners	22/28 (79%)	20/24 (83%)

ETA annotation performance, using either Match Augmentation-based ETA (MA ETA) or Paired Distance Matching-based ETA (PDM ETA), searched against the 2004 Target Set. The number of proteins in total, with matches, with at least one true match, with plurality winners, and with correct plurality winners are shown.

**Figure 2:**
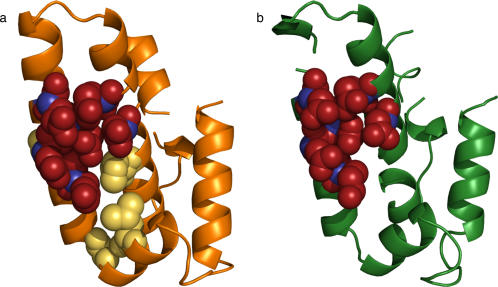
Example of Evolutionary Trace Annotation. Illustration of a source protein (2a, PDB 1yvw, chain A), its ET cluster (yellow), residues chosen as a template from that cluster (red), and the C_α_ atoms which define the geometry of the template (blue); and its functionally relevant match in a target protein (2b, PDB 2a7w, chain A), with corresponding match residues (red) and C_α_ atoms (blue).

As an example, to annotate *Bacillus cereus* phosphoribosyl-atp pyrophosphohydrolase (PDB 1yvw, chain A), ETA identifies the first cluster of 10 residues that are on the protein's surface. In this case, this occurs at the 15^th^ percentile rank. From these, ETA picks the six highest-ranked residues (39, 42, 46, 62, 43, 65; Figure 2a). The template is then the coordinates of the C_α_ atoms of these six amino acids from 1yvw and their types (K, E, E, E, E, D), allowing for variations that may occur frequently in homologs (none in this case). The PDM algorithm identifies a match with 39% sequence identity in *Chromobacterium violaceum* phosphoribosyl-atp pyrophosphatase (PDB 2a7w, chain A, EC 3.6.1; Figure 2b): six amino acids (K40, E43, E47, E63, E44, D66) with C_α_ atom distances between that each match those of their template counterparts within ±2.5 Å. Since the overall RMSD of the match (0.2 Å) is less than 2 Å, it is evaluated by the SVM, which classifies it as a significant match based on two features: the low RMSD and the similarity between the evolutionary importance of the source template residues and the matched residues (the difference is about 1 percentile rank for each pair of residues). As this is the only match found by ETA, its function achieves plurality and leads to the (correct) assignment to 1yvw of the function hydrolase activity on acid anhydrides in phosphorus-containing anhydrides (EC 3.6.1).

### Many-to-one Matching

We next asked whether a reciprocal *many-to-one* ETA matching strategy improved annotation. This reverse strategy, illustrated in Figure 1b, searches the structure of the unknown protein (S) for matches to templates (*t_i_*) derived from all the proteins with known function. The search is therefore from many *t*'s to one S, rather than from one *s* to many T's. The templates *t_i_* can be generated on a large scale and automatically since ETA relies on ET rather than experiments to extract putative determinants of a protein's function. Moreover, many-to-one and one-to-many results should be different because S and T will only produce identical templates *s* and *t* if they are close homologs. Table 2 compares many-to-one and one-to-many on the same set of 49 enzymes using an updated (2006) set of target structures (Dataset S4). Many-to-one does not improve on one-to many: the two methods have similar accuracy. Many-to-one ETA yielded 30 annotations, of which 87% were correct, whereas one-to-many ETA made 33 annotations with 85% accuracy.

**Table 2:** ETA Annotation of PSI Test Set.

	One-to-Many	Many-to-One	Reciprocal	Non-reciprocal
Proteins	49	49	49	19
With Matches	40/49 (82%)	36/49 (73%)	31/49 (63%)	12/19 (63%)
With At Least One True Match	36/40 (90%)	32/36 (89%)	30/31 (97%)	7/12 (58%)
With Vote Winners	33/40 (83%)	30/36 (83%)	30/31 (97%)	10/12 (83%)
With Correct Winners	28/33 (85%)	26/30 (87%)	30/30 (100%)	5/10 (50%)

ETA annotation performance for the PSI Test Set when searched against the 2006 Target Set, using one-to-many matching, many-to-one matching, reciprocal matching, and non-reciprocal matching.

This similarity in overall performance, however, belies important differences between the two methods, which often do not find identical matches. For example, the template extracted from *Thermus aquaticus* adenine-specific methyltransferase (PDB 1g38, chain A) matched the structure of *Escherichia coli* type I restriction enzyme ecoki m (2ar0, chain A), but the reverse was not true: the template from the restriction enzyme did not match the methyltransferase. Such asymmetry is common: out of 138 (S→{T*_i_*}) one-to-many matches and 129 ({T*_i_*}→S) many-to-one matches, only 76 matches involve identical S-T*_i_* pairs; thus one-to-may and many-to-one matches yield non-redundant information.

### Reciprocal Matching

The non-equivalence of many-to-one and one-to-many matches raises the possibility that they may be combined to increase specificity. The rationale is that in the example above, either one method has a false negative and lower sensitivity, or the other has a false positive and lower specificity. Either way, narrowing acceptable matches to only those found by both searches—that is, from *s* to T *and* from *t* to S, as shown in Figure 1c—should increase annotation specificity and accuracy, if at the cost of sensitivity.

**Figure 3:**
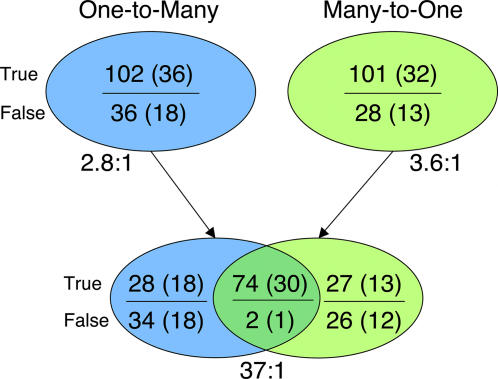
Matches to the PSI Test Set. The number of true and false matches to the PSI test set before and after reciprocal filtering is shown. The top ovals show the number of true and false matches found by each method alone, with the number of query proteins in parenthesis, and the true/false enrichment ratios below. The bottom ovals show the same data with reciprocity imposed, taking the intersection of the matches found by each method.

This hypothesis was tested by considering the *reciprocal* ETA matches at the intersection of the one-to-many and many-to-one searches. Figure 3 shows that in the control set of 49 annotated enzyme structures solved by the PSI, the former identified 102 true and 36 false matches, and the latter found 101 true and 28 false matches. Strikingly, of 76 matches common to both, 74 were true and only two were false. Thus, the true to false enrichment among reciprocal matches jumped from 3- to 37-fold. In turn, annotation accuracy rose from 85% and 87% to 100% (30 correct predictions out of 30, Table 2). This 100% accuracy does not constitute a perfect result: 19 proteins lack predictions, and ETA would necessarily miss secondary functions for “moonlighting” proteins (though no evidence suggested multiple functions). Despite this, the fact that ETA produces no erroneous annotations is remarkable.

Four observations buttress the significance of reciprocal ETA matches. First, one apparently false reciprocal match was in fact a typographical error in the PDB file of a 1-pyrroline-5-carboxylate reductase from *Streptococcus pyogenes* (PDB 2amf, chain A) [11], [81], erroneously annotated as EC 1.2.1.5, instead of EC 1.5.1.2 as per the original paper [82], elsewhere [81], and the PDB annotation of 2ahr, chain E, which is the match that led to ETA's annotation and a different structure of the *same* protein. The remaining incorrect reciprocal matches are both to one protein, 6-phosphogluconolactonase from *Thermotoga maritime* (PDB 1vl1, chain A). They appear to represent the rare case where reciprocal ETA identifies matches that are functionally divergent but structurally similar: Glucosamine 6-phosphate deaminase/isomerase NagB from *Escherichia coli* (PDB 1fs5, chain A), has the same SCOP fold as the query, while the other, a *Bacillus subtilis* hydrolase (PDB 2bkx, chain A), does not have a SCOP classification but appears to have the same fold as well.

**Figure 4:**
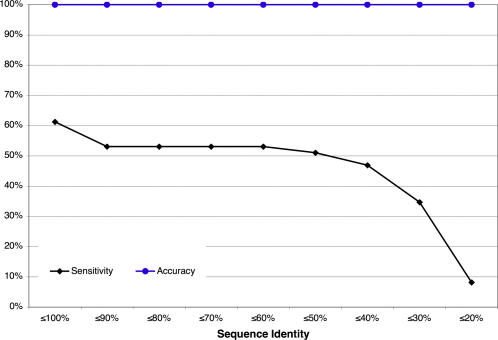
ETA and Sequence Identity. ETA performance on the PSI Test Set is shown, removing matches above a sequence identity cutoff to explore the importance of matches with varying levels of similarity. Sensitivity (black diamonds) is the percentage of the 49 proteins for which ETA predicts a correct function; accuracy (blue circles) is the percentage of these predictions that are correct.

Second, improved specificity did not lower sensitivity. Rather, the removal of some non-reciprocal, false matches enabled additional correct functions to reach plurality. Thus sensitivity rose as well (30 versus 28 or 26). Third, the case involving 2amf (discussed above) raised a concern that reciprocal ETA annotations often involved trivial high sequence identity matches. But Figure 4 shows that the increasing removal of reciprocal matches with sequence identities above a cutoff (in 10% intervals from 90% down to 20%) does not decrease accuracy. Moreover, sensitivity remained above 50%, even at the 40% threshold. Lastly, the accuracy of reciprocal ETA is in stark contrast to that of the non-reciprocally filtered matches to the remaining proteins. These yield only 49 true versus 60 false matches, which lead to ten plurality annotations with only 50% accuracy. Thus, reciprocal ETA searches are a scalable strategy to raise annotation accuracy.

These results suggest that ETA's template picking heuristics identify functionally specific amino acids. This was tested by comparing templates with PDB SITE records or Catalytic Site Atlas [67] (CSA) residues. Only one of the 49 control enzymes had a SITE record in its structure file, *Escherichia coli* ribose-5-phosphate isomerase (1o8b, chain A); it indicated a functional site of 11 residues, and the ETA template overlapped with four of them. Twenty-two of the 49 proteins also had residues noted in the CSA. In 17 cases, the CSA residues and ETA templates overlapped by an average of about two residues per protein (a third of the template or half of the CSA residues). ETA made correct reciprocal predictions in 10 of these 17 cases. In the remaining five proteins, the CSA noted only one or two residues and there was no overlap with the ETA templates. Thus, consistent with prior data [77], ETA templates fall in the neighborhood of known functional sites in all but one case, and achieve an overlap in 18 of 23 proteins that, if imperfect, is sufficient to support accurate annotation, despite having no prior experimental knowledge of the functional mechanism.

Ideally, functional similarity due to convergent evolution could be detected from template matches across folds. However, for the 18 of 30 reciprocal predictions with CATH classification [83] of both the matched structures and the templates' sources, the two were identical at all four levels: architecture, fold, super family and sequence. This may indicate that current ETA templates are not only function-specific but also structure-specific.

In summary, these enzyme controls show that ETA exploits evolutionary information to identify biologically relevant 3D templates and structurally relevant matches. Using a combination of the specificity of reciprocal ETA, which achieves the near 100% predictive accuracy, and the sensitivity of non-reciprocal ETA, which provides additional results, yields a desirable balance of sensitivity and specificity for functional annotation.

### Comparison to ProFunc Template Methods

ETA was also compared (Table 3) to two other template methods [69] from the popular ProFunc metaserver [84]. In the Enzyme Active Sites (EAS) method, templates are derived from the CSA record of functional residues. Hence, only five were available for the 49 control enzymes. The top ranked match of each of these five was correct four times (80% accuracy), resulting in low (8%) sensitivity.

**Table 3:** ProFunc Template Annotation of PSI Test Set.

	Enzyme Active Sites	Reverse Templates
Proteins	49	49
With Matches	5/49 (10%)	45/49 (92%)
With At Least One True Match	5/5 (100%)	35/45 (78%)
With Correct Top Match	4/5 (80%)	30/45 (67%)

ProFunc annotation performance for the PSI Test Set when searched against the 2006 Target Set, using either enzyme active site templates or reverse templates.

A better comparison is to the Reverse Templates (RT) method, which, like ETA, also creates templates without prior knowledge of functional sites. Unlike ETA, this is done by choosing multiple semi-random templates of just three residues, biased towards conserved, non-hydrophobic, structurally neighboring residues with minimal overlap with other chosen templates. RT identified matches for 45 of the 49 test proteins and 30 of these had a correct top-scoring match. Thus, RT is 61% (30/49) sensitive and 67% (30/45) accurate, compared to 61% (30/49) and 100% (30/30) for ETA. Notably, 27 of the predictions were common to RT and ETA. Hence, ETA made three unique predictions and all were correct, while RT made 18 unique predictions and only seven were correct; none of these could be shown to cross folds. Thus ETA is more accurate and just as sensitive.

### Negative Controls on Non-enzymes

Because ETA was specifically developed to predict enzymatic function, a risk of applying it to unannotated proteins is that it may falsely assign EC annotations to non-enzymes, which form a major part of the proteome. But Table 4 shows that reciprocal ETA did not produce a single false enzymatic annotation in 50 non-enzymes (Dataset S5) used as a negative control. In contrast, non-reciprocal matches produced 10 false enzymatic functions. Intriguingly, GO molecular function annotations were available for 36 of the non-enzyme controls, and ETA identified reciprocal matches for 27 of these in the 2006 PDB90 (Dataset S6). All yielded accurate non-enzymatic GO annotations. This suggests, first, that ETA may be applied reliably to any protein structure, enzymes and non-enzymes alike, to specifically annotate catalytic activity among the fraction that are enzymes, Second, this suggests that ETA may scale in the future to include a broader range of protein functions.

**Table 4:** ETA Annotation of Non-enzyme Set.

	One-to-Many	Many-to-One	Reciprocal	Non-reciprocal
Proteins	50	50	50	50
With Matches	12/50 (24%)	4/50 (8%)	0/50 (0%)	15/50 (30%)
With Vote Winners	8/12 (67%)	3/4 (75%)	0/0 NA	10/15 (67%)

Results of attempted ETA prediction of enzymatic functions for 50 non-enzymes.

### Positive Controls on Experimentally Annotated Enzymes

Next, to further test ETA, a prototype high-throughput hydrolase and oxidoreductase assay pipeline provided 36 enzymes annotated with EC class, subclass, and sub-subclass (the first three EC digits) [12] provided an experimental gold standard (Dataset S7). As shown in Table 5, only 11 of these proteins had known structures, and ETA made five predictions for them, all based on matches to proteins with less than 30% sequence identity. Four were clearly correct and the fifth one may be as well (*Escherichia coli* YihX, below). In addition, two more proteins without structures had close structural homologs onto which ET ranks could be mapped to extract templates: EC YbjI, with 52% sequence identity to chain A of 2hf2 (an *Escherichia coli* hydrolase); and EC YafA, with 69% sequence identity to chain A of 1nng (a *Haemophilus influenzae* hydrolase). These templates also led to correct reciprocal ETA annotations. Finally, non-reciprocal ETA led to three additional predictions; two are correct. One of these was *Thermoplasma acidophilum* TA0175 (PDB 1l6r, chain A), a hypothetical protein that had not been annotated by sequence-based methods due to low sequence identity to homologs [12].

**Table 5:** ETA Annotation of Toronto Set.

	One-to-Many	Many-to-One	Reciprocal	Non-reciprocal
Proteins	13	13	13	6
With Matches	8/13 (62%)	13/13 (100%)	7/13 (54%)	5/6 (83%)
With At Least One True Match	6/8 (75%)	9/13 (69%)	6/7 (86%)	2/5 (40%)
With Vote Winners	7/8 (88%)	11/13 (85%)	7/7 (100%)	3/5 (60%)
With Correct Winners	6/7 (86%)	9/11 (82%)	6/7 (86%)	2/3 (67%)

Results of ETA Annotation of recent experimentally annotated enzymes.

**Figure 5:**
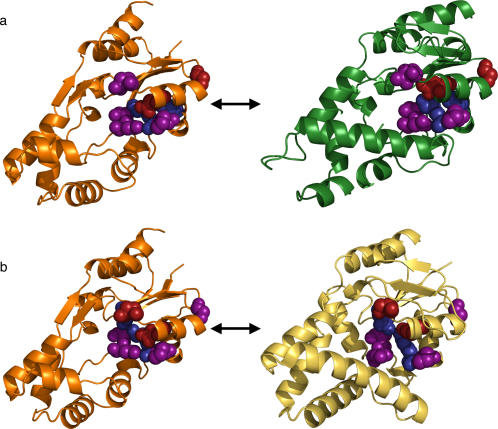
EC YihX and Matches. Comparison of structures and template/match residues for query 2b0c, chain A (4a and 4b, orange), from the Toronto Set versus targets 1×42, chain A (4a, green), and 1zrn (4b, yellow). Purple spheres, residues in both the source and target template and match; red spheres, residues in only the query template and target match; blue spheres, residues in only the target template and query match.

The questionable annotation mentioned above involved *Escherichia coli* YihX (Swiss-Prot P32145; PDB 2b0c, chain A) predicted by ETA to be a phosphatase that hydrolyzes halide bonds in c-halide compounds (EC 3.8.1). The evidence came from two reciprocal matches to remote homologs with similar folds (1×42, chain A and 1zrn, at 22% and 20% sequence identity, respectively, shown in Figure 5). This prediction concurred with several other sources (InterPro [85], PRINTS [86], and TIGERFAMs [87]) that classify this protein as a haloacid dehalogenase-like (HAD-like) hydrolase. These proteins frequently also carry phosphatase activity [12], consistent with the experimental assay, which suggested phosphoric monoester hydrolase activity (EC 3.1.3) as a function. The experimental essays did not, however, test for the function predicted by ETA. Thus one strong possibility may be that the experimental annotation is incomplete rather than in conflict with ETA's prediction.

In summary, despite the small number of structures available, predictions are available for 10 of 13 proteins. Eight were clearly correct while one additional prediction (EC YihX) may be as well. Seven predictions arose from reciprocal ETA, which is at least 86% (6 of 7) accurate, including two predictions based on homology models of EC YbjI and YafA. These last two annotations further suggest that the scope of reciprocal ETA annotations can extend to proteins with structural homologs—and thus expand beyond the structural proteome.

### Predictions for Structural Genomics Proteins

Following these small-scale studies, we next tested whether ETA could predict function over the entire structural proteome, following other efforts [88]–[90]. First, conveniently, 1314 SG proteins already annotated with 3 or 4 digit EC numbers provided a large-scale positive control. Of these, 1218 (93%, Dataset S8) had enough homologs to support ET analyses. ETA predicted functions for 517 that agreed with prior annotations in 478 cases (92% accuracy, Table 6). This suggest an 8% misannotation rate (39 disagreements) although some of these may also be due to incomplete or incorrect annotations. Of note, among the 701 other proteins, non-reciprocal ETA suggested functions in an additional 407, 291 of which agreed with prior annotations (71% accuracy). Thus the large-scale accuracy of reciprocal ETA remains above 90%, but non-reciprocal matches can still make a non-negligible contribution.

**Table 6:** ETA Annotation of Structural Genomics Annotated Set.

	One-to-Many	Many-to-One	Reciprocal	Non-reciprocal
Proteins	1218	1218	1218	701
With Matches	914/1218 (75%)	745/1218 (61%)	527/1218 (43%)	494/701 (70%)
With At Least One True Match	801/914 (88%)	614/745 (82%)	486/527 (92%)	378/494 (77%)
With Vote Winners	837/914 (92%)	659/745 (88%)	517/527 (98%)	407/494 (82%)
With Correct Winners	716/837 (86%)	547/659 (83%)	478/517 (92%)	291/407 (71%)

Results of ETA annotation performance for annotated structural genomics proteins.

**Figure 6:**
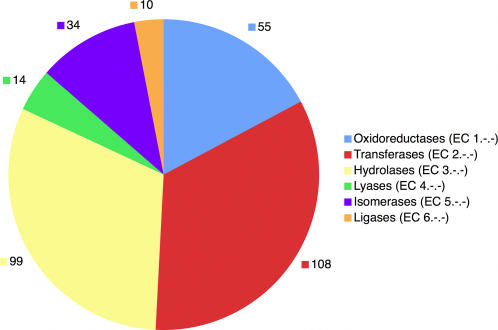
EC Classes of ETA Predictions. Distribution of 320 reciprocal ETA annotations among the first digit EC classes, including both first and second order predictions.

ETA was then applied to make genuine predictions of enzymatic function among the remaining 3114 SG proteins that lack any annotated catalytic activity. The 2935 (94%, Dataset S9) that were amenable to ET analysis lead to 258 enzymatic annotations, as shown in Table 7. These fell in the six EC classes in proportions that were within 6% of those for all PDB90 proteins, as shown in Figure 6. While the availability of predictions is low (9%), we note first that many of the 2935 proteins are likely to be non-enzymes, for which the lack of enzymatic activity prediction is a desirable outcome. Thus the actual availability of predictions for enzymes should be higher. Second, the preceding computational controls suggest that most of the 258 predictions will prove correct. Third, 20 proteins were already partially annotated with 1 or 2 EC digits, and 19 of these are in agreement with ETA annotations.

**Table 7:** ETA Annotation of the Structural Genomics Unannotated Set.

	One-to-Many	Many-to-One	Reciprocal	Non-reciprocal
Proteins	2935	2935	2935	2677
With Matches	1027/2935 (35%)	553/2935 (19%)	269 (334*)/2935 (2935*) (9%) (11%*)	933/2677 (35%)
With Vote Winners	827/1027 (81%)	484/553 (88%)	258 (320*)/269 (334*) (96%) (96%*)	706/933 (76%)

Summary of ETA annotation of unannotated structural genomics proteins. For detailed information see the supplementary materials. ^*^These numbers include second-order predictions.

The one ambiguity is *Becilius cereus* BC_3378 (PDB 2b81, chain A) that is annotated as an oxidoreductase acting on paired donors with incorporation or reduction of molecular oxygen (EC 1.14.-). However, ETA suggested an oxidoreductase acting on the CH-NH group of donors with other acceptors (EC 1.5.99). based on one reciprocal match to *Methanosarcina barkeri* coenzyme F420-dependent methylenetetrahydromethanopterin (PDB 1z69; chain A), which had 21% sequence similarity to the source protein. Thus the two annotations agree on oxidoreductase activity, but disagree on the donor group. This error on the part of ETA arises from a known global structural similarity between bacterial luciferases (such as the query protein) and its methylenetetrahydromethanopterin match [91]. Thus ETA identifies a meaningful local structural similarity, but not one specific enough to indicate functional similarity to two EC digits of precision. In all 20 cases, though, ETA identifies functionally relevant similarities, 95% of which are entirely consistent with existing partial annotations.

To determine the degree to which these 258 reciprocal predictions were novel, they were also compared with ProFunc annotations. In 167 proteins, ProFunc's annotations agreed completely with ETA's. The remaining 91 predictions are unique to ETA. For 36 proteins, the methods differ at the first, second, or third EC digit (7, 24, and 5 proteins, respectively). In 24 proteins, ETA offers more specific predictions than ProFunc, which produces only one or two EC digits in these cases (6 and 18 proteins, respectively); these agree with ETA. For 31 proteins, ProFunc offers no prediction (8 proteins), predicts only “enzymatic activity” (2 proteins), or predicts only non-enzymatic functions (21 proteins). It is important to emphasize here that ProFunc incorporates approaches beyond 3D templates, including four template-based methods, five sequence-based methods, and five global structure-based methods. Thus, ETA may prove even more useful in combination with other methods.

Intriguingly, it appears to be possible to apply ETA iteratively to make additional predictions. First, the 258 reciprocal annotations were added to the target set of annotated proteins, and ETA was repeated on the 2677 that remained without function. With this second pass, ETA added nearly 25% (62) more predictions: 52 previously based on non-reciprocal matches, plus 10 completely novel ones. Likewise, annotation from non-reciprocal matches increased 14% (96). Thus such second order predictions significantly raise the sensitivity of 3D template annotations for structural genomics.

### Molecular Analysis of Predictions

**Figure 7:**
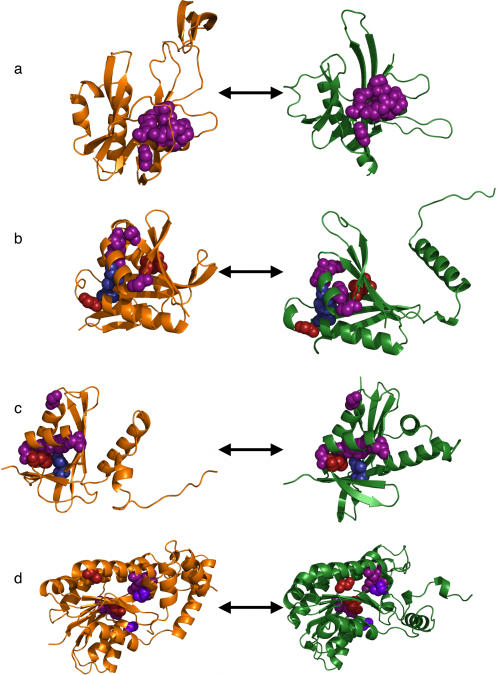
Examples of ETA Predictions. Reciprocal matches contributing to three novel ETA function predictions, with the query in orange and the target in green, and template/match residues using the scheme in Figure 5. 7a, query 1jrk, chain A, vs. target 1vhz, chain B; 7b, 1wwz, chain B, vs. 1y9w, chain A; 7c, 2fl4, chain A, vs. 1wwz, chain B; 7d, 1xkq, chain A, vs. 1jtv, chain A.

In order to clarify the meaning of these predictions, a few were examined in detail. The first example demonstrated functional annotation in the “twilight zone” of sequence identity. Four of five reciprocal ETA matches suggested that PAE3301 from *Pyrobaculum aerophilium* (PDB 1jrk, chain A) was a hydrolase acting on phosphorus-containing acid anhydrides (EC 3.6.1), a prediction unique to ETA versus ProFunc. Remarkably, sequence identities between the source and targets were between 16% and 25%, so no matches are to close sequence homologs. Moreover, the template match to one of them, the *C. elegans* ap4a hydrolase binary complex (16% sequence identity, PDB 1vhz, chain B, Figure 7a), was especially revealing because it overlapped six residues (underlined) of the GX_5_EX_7_ REUXEEXGU motif [92] (X: any residue; U: I, L, or V) associated with the EC 3.6.1 activity in the target protein [93]. Interestingly, the *Pyrobaculum* sequence deviates slightly from this motif, with an F at the position of the first U.

The second example demonstrated iterative annotation. On the one hand, EF_1086 (*Enterococcus faecalis*, PDB 2fl4, chain A) had three matches suggesting it was an acyltransferase that transfers groups other than amino-acyls (EC 2.3.1); however none of these matches were reciprocal. On the other hand, ETA predicted this same function for PH1933 (from *Pyrococcus horikoshii* OT3, PDB 1wwz, chain B) based on two reciprocal matches: one to an acetyltransferase from *Bacillius cereus* with 15% sequence identity (PDB 1y9w, chain A, Figure 7b), and the other to a phosphinothricin acetyltransferase from *Agrobacterium tumefaciens* with 24% sequence identity (PDB 1yr0, chain A). Once this second, independent result was fed back into the target set, it reciprocally matched 2fl4 (Figure 7c), with which it shared 25% sequence identity, and led to the EC 2.3.1 annotation of EF_1086.

The last example reinforces the functional role of template residues. ETA identified 21 reciprocal matches with sequence identities varying between 19% and 65% for R05D8.7 (*Caenorhabditis elegans,* PDB 1xkq, chain A). Nearly all these matches (19) concur on the predicted function, suggesting oxidoreductase activity acting on CH-OH group of donors with NAD or NADP as acceptor (EC 1.1.1), another unique prediction compared to ProFunc. One of the matches is to a human 17beta-hydroxysteroid dehydrogenase type 1 (Figure 7d, PDB 1jtv, chain A) with 21% sequence identity, and it involved three of the five catalytic residues suggested for 1jtv by the CSA. Two (Y155 and K159 in 1jtv) were represented in both the reciprocal template of the target and the source template (Y162 and K166 in 1xkq). One additional residue (S142) was unique to the reciprocal template and matched the source (S148). This underscores that here, as with prior controls, ETA annotation is reliable because its templates and matches involve functionally significant residues.

All predictions are available as supplementary data (one-to-many predictions, Dataset S10; many-to-one predictions, Dataset S11; reciprocal predictions, Dataset S12; second-order reciprocal predictions, Dataset S13; non-reciprocal predictions, Dataset S14).

### Conclusions

This study aimed to transfer functional annotations between protein structures based on the local structural and evolutionary similarities of their functional sites. This was made possible through the automated ET analysis of functionally important residues [71] and substantial increases in the computational efficiency of geometric matching. As a result, an ETA pipeline could perform both one-to-many and many-to-one template searches to identify reciprocal matches. Combined with plurality voting [76], selecting reciprocal matches stringently removes false positives and increases specificity so as to yield reliable annotations in positive, negative, experimental, and large scale controls that improve on existing template methods [69]. Thus ETA suggested 258 enzymatic function predictions (plus an additional 62 through iteration) of high predicted reliability (over 90%) in the structural proteome, of which 91 are unique to ETA over the ProFunc metaserver. These should lead to efficient and systematic use of appropriate assays for experimental annotation [12]. An ETA server will be available on the ET server web site at http://mammoth.bcm.tmc.edu.

While this work focused on enzymatic annotation, a preliminary examination of GO predictions on these same proteins produced correct annotations. This suggested that ETA might be extended to non-enzymes, consistent with the many experiments where ET guided the functional redesign of non-enzymes [74], [75], [94]. Likewise, preliminary use of homology modeling suggested that 3D template annotations could extend beyond the currently limited structural proteome to include its homology-modeled neighborhood. Both are fertile areas for future studies.

Notably, ETA compares well to other template methods—both those that rely on experimentally determined catalytic sites, and those that derive templates via computational means. ETA had significantly higher (7x) sensitivity than ProFunc's Enzyme Active Site method, which relies on known catalytic sites. Compared to ProFunc's Reverse Templates method which does not depend on such knowledge, ETA is just as sensitive (61%) but significantly more accurate (100% vs. 67%).

The origin of this significant improvement is not likely to be due to differences in structural matching techniques; rather, ETA templates and their matches must be more functionally relevant as a result of two techniques unique to this work. First, ETA templates are defined with ET, which identifies and ranks residue variations that trigger major evolutionary divergences. Since divergences involve evolutionary trees, ET ranks differ from other measures of “conservation”, and a growing body of experimental evidence suggests that top-ranked ET residues clustered on the surface are important determinants of function [72], [74], [75], [94]–[96]. Thus ET ranks should lead to more precise approximations of active sites. Indeed, controls presented here confirm that ETA templates frequently overlap known active sites. Also, past work showed that pinpoint identification of the active site was not essential as long as the template consisted of important residues near the active site [76], [77].

Second, the ETA pipeline strives to raise specificity. It is important to note the emphasis here on annotation specificity, as misannotations may propagate and prove difficult to eradicate from all databases. In particular, the massive number of false positive geometric matches to a C_α_ template easily overwhelms the few true positives. ETA thus applies three orthogonal and successive filtering steps: the requirement that the matched site residues have similar ET ranks as the template; the requirement that a match from one protein to another be reciprocated, exploiting the complementary information in both searches; and the requirement that a plausible annotation of function achieve a plurality of votes through more matches than any other alternative. These three requirements each individually raise the stringency of annotation, but when combined they drastically reduce the likelihood that an annotation is due to random chance, as shown by the lack of false enzymatic annotations on the non-enzyme negative controls.

More broadly, there are now many computational annotation methods based on identifying different types of similarity between proteins. Pooling this information can be especially useful, as shown by meta-servers such as ProFunc [84] and JAFA [97], and by graph theoretic methods [98], [99]. Further improvements should be expected as more inconsistencies are identified and excised not only among methods but also within individual ones. The latter point was demonstrated here by imposing consistency between matches, which leads to plurality, and between one-to-many and many-to-one 3D template searches, which leads to reciprocity. This highlights the complex nature of measures of functionally relevant similarities in proteins. Each alone may not be reliably meaningful or reproducible, but requiring post hoc consistency among them can richly increase functional prediction specificity with, as here, little if any loss of sensitivity.

## Materials and Methods

### Function Definition

Here, two proteins are considered to have the same function if they share the first three digits of their EC numbers, as the fourth digit represents a serial number assigned to each distinct enzyme in that section of the hierarchy and does not carry a consistent functional meaning [100]. Additionally, high throughput experimental methods offer this level of precision [12]. EC numbers for proteins of known function were those from the proteins' PDB files, except for proteins from the Toronto functional annotation pipeline, whose annotations were taken from that publication [12].

### Data Sets

The “Training Set” (Dataset S1) is the set of 53 enzymes used previously [77] to train the SVM and to choose values for the distance tolerance parameter ε and the RMSD cutoff in this study (see below).

The “PSI Test Set” (Dataset S3) is the same as the “PSI Set” set used previously [76], and comprises 49 annotated enzymes chosen randomly from the PSI that do not overlap with the Training Set.

The “Non-enzyme Set” (Dataset S5) is composed of 50 randomly chosen proteins from the PDB that appear to be non-enzymes. Their functions include structure, DNA and RNA binding, signaling, and oxygen transport.

The “Toronto Set” (Dataset S7) consists of 36 enzymes annotated by automated experimental screening [12], among which 11 have BLAST hits to structures in the PDB with 99% or higher sequence identity. Twenty-three proteins did not have structures, and two did not have successful ET analyses. Two of the proteins that did not have structures did have close homologs with greater than 50% sequence identity and were examined further (see “Results and Discussion”).

The “Structural Genomics Set” contains proteins with the keywords “structural genomics” or “unknown function” in the PDB [11]. There were 4372 such proteins in the PDB, 4253 of which also had ET results. EC numbers and GO terms listed in the PDB were used to identify PSI proteins annotated as enzymes, with GO terms converted to EC numbers using the EC to GO mapping [13]. There were 1218 proteins annotated to 3 or more EC digits; these are the “Structural Genomics Annotated” set (Dataset S8), and the remaining 2935 are the “Structural Genomics Unannotated” (Dataset S9) set.

The “Target Set” (Dataset S4) was the subset of the 2006 PDB-SELECT-90 [101] with ET results and single EC annotations complete to the third or fourth digit in their PDB files. This set contains 3069 proteins. Non-enzymes were also searched against 5827 traced PDB90 proteins without EC annotations. To compare PDM ETA with MA ETA, we also used an older target set of 2779 proteins from the 2004 PDB-SELECT-90 (Dataset S2) with single annotations complete to the fourth digit.

The PDB codes and protein names for each set, as well as predictions for the unannotated structural genomics proteins, are available as supplementary data.

### Template Creation

Templates were created as described elsewhere [76]. Briefly, proteins were traced using automated [102], real-valued [103] ET [70] to determine their residues' relative evolutionary importance. Residues were added in order of importance to form a structural cluster (each residue has a non-hydrogen atom within 4 Å of another residue in the cluster) of at least 10 surface residues (solvent accessibility of at least 2 Å^2^ calculated by DSSP [104]), and the six most important are chosen. Ties were broken by choosing the residue closest to a point halfway between the centroid of the cluster residues and the centroid of the current template residues. Residues are represented geometrically by their C_α_ atoms. The residue types of matched positions must be a combination seen more than once in the ET multiple sequence alignment.

For the two Toronto Set proteins modeled with homologous structures, ETA applies ET to the sequence of the query protein—including the homologous structure in the alignment but not in the calculation of ET results—and maps the residue types and ET results to the structure using the multiple sequence alignment. Only non-gap positions in the query were allowed for the template.

To demonstrate functional relevance, templates were compared to SITE records or Catalytic Site Atlas residues as of October 2007.

### Template Searching

Template searching is performed using Paired Distance Matching. Starting with residue *r_1_* in a template **R** = {*r_i_*}, PDM identifies all residues of type *t_1_* in the target protein. For the first iteration, each of these is a possible match *m_i_* to the template, and each is stored in the set **M** = {*m_i_*}.

For residue *r_2_*, all residues of type *t_2_* are identified. Each new residue is added combinatorically to each of the possible matches *m_i_* in **M**, expanding **M**. Each *m_i_* is then checked against distance constraints and retained or discarded. The distance between the new residue *r*_2_ and the old residue *r_1_* is computed; in this case distance *d(r_1_, r_2_)*. For each *m_i_*, the corresponding distances between the new residue *r_2_*′ and the residues in the current *m_i_* are computed and compared; in this case the distance of the corresponding matched residues *d*(*r_1_*′, *r_2_*′) is compared to *d*(*r_1_*, *r_2_*). The match is removed if *|d*(*r_1_*, *r_2_*)-*d*(*r_1_*′, *r_2_*′)*|*≥ε; where ε represents a tolerance value; otherwise *m_i_* remains in **M**.

These steps are repeated for *r_3_*, with each residue of type *t_3_* in the target added to each *m_i_*, distances *d*(*r_2_*, *r_3_*), and *d*(*r_1_, r_3_*) computed and compared to their counterparts in *m_i_*, and each *m_i_* with all distances within ε of the template distances retained in **M**. This process continues for each remaining template residue *r_i_*, halting when **M** becomes empty or all residues in the template have been examined. The result is a set of matches whose distances between residues match those of the original template plus or minus ε. If the distances match, the residues in *m_i_* are likely in a similar geometry to those in *R*, so the residue numbers of each *m_i_* are reported with their RMSD.

ε is set at 2.5 Å. Values from 1 to 6 in 0.5 Å steps were tested on the Training Set; 2.5 represented the best balance of post-SVM positive predictive value and sensitivity in identifying true matches.

For one-to-many matching, templates were created for the query protein and searched against the 2006 Target Set unless noted otherwise. For many-to-one matching, templates were created for the Target Set proteins and then searched against the query protein (excepting 13 backbone-only structures with no solvent accessibility data).

### Match Filtering

Three filters removed likely false matches. First, matches with an RMSD greater than 2 Å were eliminated. Values from 1 to 5 in increments of 0.5 Å were tested for matching performance; of these, 2 Å was the best compromise between sensitivity and positive predictive power (as in the ε optimization). Consistent with this, true matches are rare beyond 2 Å.

Next, an SVM filters additional matches based on geometric and evolutionary similarity. The SVM feature vector is seven dimensional, made up of match RMSD, which quantifies geometric similarity (1 dimension), and the sorted absolute values of the difference between the percentile ET ranks of each pair of matched residues, which quantifies evolutionary similarity (6 dimensions). The SVM was created with the Spider package for MATLAB (http://www.kyb.tuebingen.mpg.de/bs/people/spider), using a balanced ridge set to the difference in the proportions of true and false matches, a radial basis function kernel with the parameter σ = 0.5, and all other parameters left at default values. Training was performed using matches from the Training Set against the 2004 Target Set and four digits of EC precision. SVMs trained using the 2006 PDB-SELECT-90 and 3 digit precision were evaluated but did not significantly change classification. For more about the SVM, see [76], [77].

Finally, reciprocal ETA removes non-reciprocal matches, taking only those in the intersection of the sets of matches found by the two matching methods.

### Voting

Each remaining match, excluding self-matches, represents one vote for its annotated function, and this set of functions represents possible annotations. The function achieving a plurality of votes wins. A protein counts only once per query. No single prediction is made when no plurality is reached (a tie); instead ETA offers multiple possible annotations.

Voting was performed using the set of many-to-one matches, one-to-many matches, the intersection of these two sets (reciprocal ETA), or the union of these two sets (non-reciprocal ETA). Non-reciprocal predictions are made when reciprocal predictions are not available, which can occur due to a lack of matches or a tie vote.

### Sequence Identity

Sequence identity between pairs of proteins was calculated on global alignments produced by CLUSTALW [105] with its default settings.

### Comparisons to ProFunc

ProFunc results for the Enzyme Active Sites templates, Reverse Templates, and all methods combined are those provided by the ProFunc web server. For the template method comparisons, this meant that only the top five matches were given (which frequently included a self-match; these were removed). Additionally, proteins are matched against the entire PDB, raising concerns about redundant matches. This was ignored for EAS due to the small number of matches found, but because RT generally found more matches, those results were restricted to proteins found in our PDB90 target set to limit redundancy and ensure that the comparison showed differences between the two methods' performance, rather than their target data sets. The RT method sometimes identified proteins with no enzymatic annotations; these were considered false predictions. ETA's structural genomics functional predictions were compared to those of ProFunc by taking the ProFunc server's predicted functions and manually mapping them to EC numbers.

All ProFunc results were retrieved in October 2007, except for EAS results for the 49 proteins, which were retrieved in December 2007.

### Visualization

Images of templates and matches were generated using PYMOL [106].

## Supporting Information

Dataset S1 The set of 53 enzymes used previously to train the SVM and to choose values for the distance tolerance parameter ε and the RMSD cutoff in this study (see below).
(0.00 MB TXT)

Dataset S2 To compare PDM ETA with MA ETA, also we used an older target set of 2779 proteins from the 2004 PDB-SELECT-90 with single annotations complete to the fourth digit.
(0.04 MB TXT)

Dataset S3 Comprises 49 annotated enzymes chosen randomly from the PSI that do not overlap with the Training Set.
(0.00 MB TXT)

Dataset S4 The “Target Set” was the subset of the 2006 PDB-SELECT-90 with ET results and single EC annotations complete to the third or fourth digit in their PDB files. This set contains 3069 proteins.
(0.05 MB TXT)

Dataset S5 Composed of 50 randomly chosen proteins from the PDB that appear to be non-enzymes. Their functions include structure, DNA and RNA binding, signaling, and oxygen transport.
(0.00 MB TXT)

Dataset S6 Non-enzymes were also searched against 5827 traced PDB90 proteins without EC annotations.
(0.03 MB TXT)

Dataset S7 Consists of 13 enzymes annotated by automated experimental screening, among which 11 have BLAST hits to structures in the PDB with 99% or higher sequence identity, and two of the proteins have close homologs with greater than 50% sequence identity.
(0.00 MB TXT)

Dataset S8 The “Structural Genomics Set” contains proteins with the keywords “structural genomics” or “unknown function” in the PDB [11]. There were 4372 such proteins in the PDB, 4253 of which also had ET results. EC numbers and GO terms listed in the PDB were used to identify PSI proteins annotated as enzymes, with GO terms converted to EC numbers using the EC to GO mapping. There were 1218 proteins annotated to 3 or more EC digits; these are the “Structural Genomics Annotated” set.
(0.02 MB TXT)

Dataset S9 The “Structural Genomics Set” contains proteins with the keywords “structural genomics” or “unknown function” in the PDB. There were 4372 such proteins in the PDB, 4253 of which also had ET results. EC numbers and GO terms listed in the PDB were used to identify PSI proteins annotated as enzymes, with GO terms converted to EC numbers using the EC to GO mapping. There were 1218 proteins annotated to 3 or more EC digits; these are the “Structural Genomics Annotated” set, and the remaining 2935 are the “Structural Genomics Unannotated” set.
(0.02 MB TXT)

Dataset S10 ETA predictions for structural genomics proteins using the one-to-many matching method. Proteins with no prediction listed had matches but no function achieved plurality.
(0.01 MB TXT)

Dataset S11 ETA predictions for structural genomics proteins using the many-to-one matching method. Proteins with no prediction listed had matches but no function achieved plurality.
(0.01 MB TXT)

Dataset S12 ETA predictions for structural genomics proteins using reciprocal matching. Proteins with no prediction listed had matches but no function achieved plurality.
(0.00 MB TXT)

Dataset S13 Reciprocal ETA predictions for structural genomics proteins using previous reciprocal predictions as target data. Proteins with no prediction listed had matches but no function achieved plurality.
(0.00 MB TXT)

Dataset S14 ETA predictions for structural genomics proteins using non-reciprocal matching. Proteins with no prediction listed had matches but no function achieved plurality.
(0.01 MB TXT)
